# Immersive virtual reality for improving flood evacuation behaviour and self-efficacy

**DOI:** 10.4102/jamba.v17i1.1655

**Published:** 2025-02-05

**Authors:** Furqan I. Aksa, Muhammad Ashar, Heni W. Siswanto, Zaidan Z. Malem

**Affiliations:** 1 Department of Geography Education, Faculty of Education, Universitas Samudra, Langsa, Indonesia; 2 Department of Informatic Engineering, Faculty of Engineering, Universitas Negeri Malang, Malang, Indonesia; 3 Center for Educational Research, Faculty of Social Sciences and Humanities Research Organization, National Research and Innovation Agency (BRIN) Republic of Indonesia, Jakarta, Indonesia

**Keywords:** immersive virtual reality, flood evacuation, behaviour, disaster, self-efficacy

## Abstract

The series of evacuation drills implemented for flood disasters were mostly through monotonous traditional pedagogical methods. The application of these methods was observed to be ineffective because of the inability to realistically represent the actual dangers and have a significant impact on behavioural changes. Therefore, this research aimed to develop and test the effectiveness of Immersive Virtual Reality (IVR) Flood Evacuation to improve knowledge and self-efficacy regarding flood disasters. This was achieved through the adoption of a one-group pretest-posttest design as well as the selection of 45 students as participants. The results showed that IVR significantly improved the knowledge of the best evacuation practices and self-efficacy (*T*-test, *p* < 0.005) with long-term influence on the memory of the participants. These observations supported the previous studies that reported the ability of virtual reality in ensuring longer knowledge retention compared to traditional learning methods.

**Contribution:**

Immersive virtual reality was found to have the potential to be applied as an interesting pedagogical tool for flood evacuation training. The application of the method for drills was discovered to be more efficient, cost-effective, and provide enhanced knowledge retention for users. This research shows the significance of seamlessly incorporating knowledge with flood evacuation practices through IVR in disaster education programmes. The integration is important in the transformation of knowledge into actionable steps, thereby enhancing overall preparedness.

## Introduction

Flood has been identified as the disaster with the most prevalence throughout the world (Kim & Gim 2020; Martins & Nunes 2020). This was confirmed by the World Disaster Report (2016) that flood was ranked in the top position among the most prevalent disasters in the world with 43% occurrence. The records from Indonesia also showed the existence of 1312 incidents in 2019 which led to 367 fatalities, 1385 injuries, and affected 649 659 people (BNPB 2019). These numbers are expected to increase because of global climate change, population growth in flood-prone areas, changes in land use and extreme rainfall (Alonso Vicario et al. 2020).

The risk of flood disasters can be reduced through effective early evacuation (Ahmadi, Karampourian & Samarghandi 2022). The concept of evacuation has been defined as a key strategy to minimise the impact of flood disasters (D’Amico et al. 2023). Moreover, emergency evacuation is considered important in quickly and effectively relocating residents from high-risk areas to safer places (Ahmadi et al. 2022) in order to protect humans, animals, historical sites and documents.

The flood evacuation behaviour capacity can be enhanced through drills and simulations (Aksa 2022). However, the traditional pedagogical methods which are normally used up to the present time have been discovered to be monotonous, thereby leading to a reduction in the interest of students to participate in the process (Mitsuhara et al. 2013). The ineffectiveness of these traditional methods is further reinforced by the fact that the current sets of students are part of the digital generation (Çoban & Göktaş 2022). The drills were discovered not to be realistic in representing the actual dangers, thereby not causing any behavioural change (Feng et al. 2020). Another point is that these traditional evacuation drills require significant resources and funding.

The challenges identified led to the need for new pedagogical methods to attract the interest of several people, specifically teenagers. An example is the application of immersive virtual reality (IVR) technology in the form of software and interactive devices to create a 3D simulation environment (Alene et al. 2023; Fromm et al. 2021). Immersive virtual reality consists of important elements such as virtual environment (VE), and immersive and interactive spaces. Moreover, it has been previously defined as a digital space where users can engage, interact and experience (Alene et al. 2023; Fromm et al. 2021).

Virtual reality (VR) is more efficient and cost-effective than traditional pedagogical approach to be implemented for flood evacuation drills (Nguyen, Jung & Dang 2019). This is because of the ability of the technology to allow users to navigate through the challenging process of subconscious thoughts using large amounts of information (Nguyen et al. 2019). Virtual reality provides an engaging learning experience for students and enhances the effectiveness of disaster education. Moreover, it simulates the dangers and threats more realistically compared to traditional learning methods in order to provide improved knowledge retention for users (Feng et al. 2020).

Several VR technologies have been applied in disaster education such as the immersive VR developed for fire safety (Lovreglio 2020) as well as earthquake and tsunami evacuation training (Lovreglio et al. 2022). Some previous studies also proved the effectiveness of these pedagogical methods in enhancing the capacity of students for disaster risk reduction. However, limited research has been conducted on the effectiveness of IVR flood evacuation training. This research was conducted to fill the gap by determining the effectiveness of IVR in improving the knowledge and self-efficacy of students regarding flood disasters.

The assessment of self-efficacy is important because of its definition as the belief in one’s ability to mobilise cognitive resources and actions required to fulfil specific situational demands (Dasci Sonmez & Gokmenoglu 2023; Hendriks & Stokmans 2020). According to the social cognitive theory for hazard response, self-efficacy is explained as a key mechanism normally used by people to regulate behaviour in the face of danger (Justice et al. 2021; Liu et al. 2023). Several previous studies have reported that people with high self-efficacy had better disaster risk reduction behaviours than those with lower self-efficacy (Martin et al. 2009). Similarly, a qualitative research on adult Mandarin-speaking migrants in the USA showed that self-efficacy played an important role in the effectiveness of people seeking help during disasters (Yip et al. 2013).

### Immersive virtual reality flood evacuation

The IVR flood evacuation developed was aimed at providing students with the opportunity to experience a flood disaster and evacuate quickly and accurately. The development process was based on the flood evacuation guidelines formulated by the National Disaster Management Agency (BNPB) of Indonesia. The guidelines consist of several behavioural responses such as knowing the location of flood evacuation points, preparing a disaster readiness bag containing food, medicines, and a flashlight needed for 3 days, storing important documents, and turning off all electrical networks. The focus of this research was on reasonable behavioural responses primarily associated with indoor or home scenarios.

**FIGURE 1:**
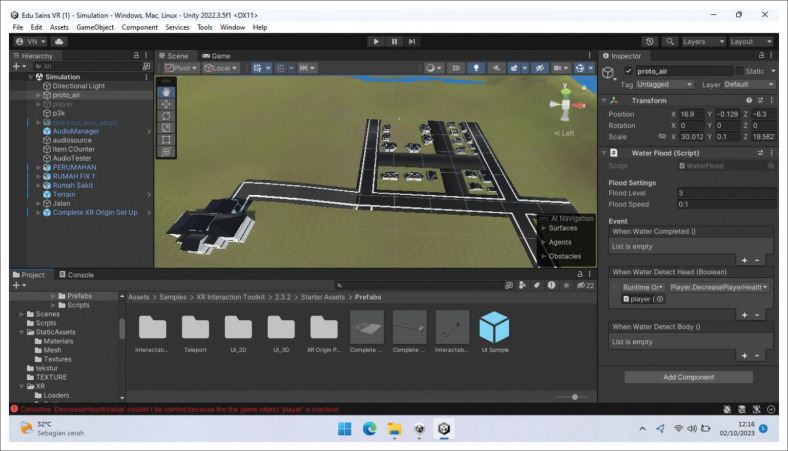
Flood evacuation area landscape.

**FIGURE 2:**
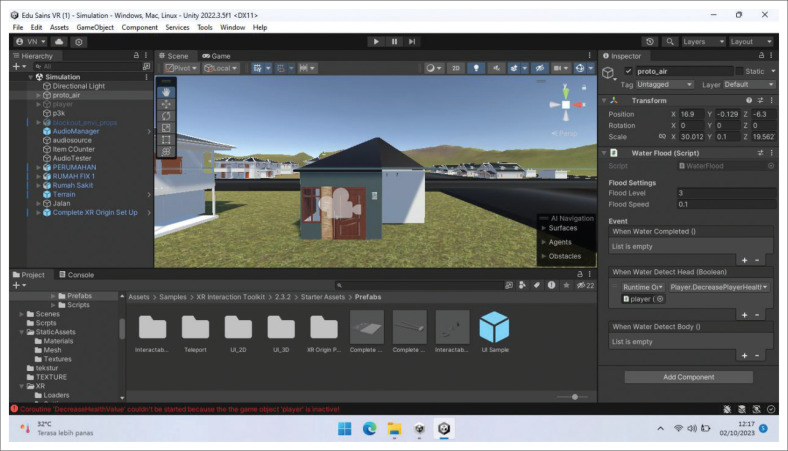
Flood evacuation drill home area.

**FIGURE 3:**
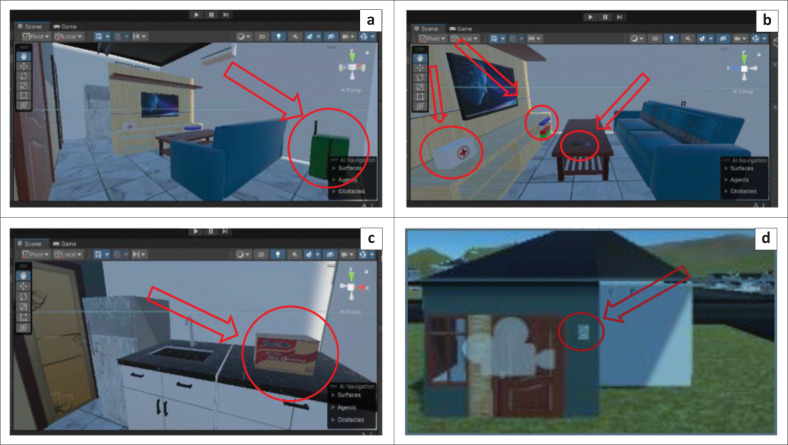
(a) Disaster readiness bag, (b) Emergency kit, (c) Food supplies, (d) Electrical network panel.

**FIGURE 4:**
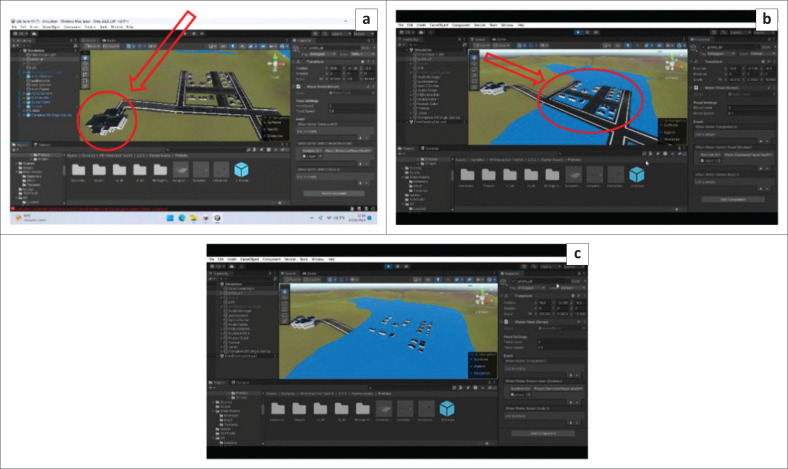
(a) Assembly point for flood evacuation, (b) Residential location, (c) Illustration of flood innudation.

**FIGURE 5:**
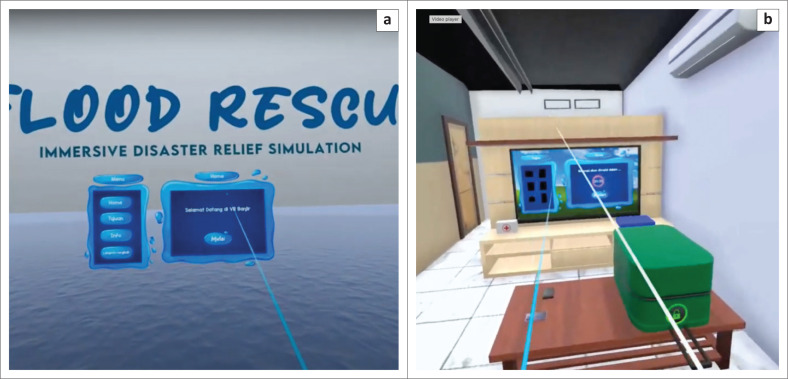
Starting interface of immersive virtual reality flood evacuation program: (a) Home page view Immersive Virtual Reality flood evacuation program, (b) Display instructions for users before starting the Immersive Virtual Reality flood evacuation.

The storyline for the flood evacuation training was set at approximately 15 min, and the participants were asked to evacuate to a gathering point during flood disaster. The scenarios designed include: (1) turning off all electrical networks before evacuation, (2) carrying a disaster preparedness bag containing food for 3 days, medicines, and a flashlight, as well as (3) not crossing swift currents, drainage channels, puddles, flooded areas, and not touching electrical equipment during the evacuation (see Figure 3a, Figure 3b, Figure 3c and Figure 3d). The game is designed to fail when participants do not reach the assembly point within 15 min. Immersive virtual reality is designed using the Unity application. The development of IVR flood evacuation begins with creating 3D assets consisting of flood evacuation area landscape, flood evacuation drill home area, resident location, and assembly point (see Figure 1, Figure 2, Figure 4a, Figure 4b and Figure 4c). After that, the immersive virtual reality product is ready to be used as shown in Figure 5a, and Figure 5b.

## Research methods and design

### Research design

This research was conducted using a one-group pre-test and post-test design. The pre-test measurements were applied before the training was provided, while the post-test measurements were applied after the implementation of IVR flood evacuation. A total of 45 students, including 17 males and 28 females, from the Geography Education class of Universitas Samudra located in Langsa City, Aceh Province, Indonesia were selected as participants. This school was selected because of the high flood risk index of the area. Moreover, the disaster risk analysis conducted by the BNPB of Indonesia showed that the population at risk of being affected by floods in the area was 125 107 people, and the estimated economic loss was 14 140 billion (Badan Nasional Penanggulangan Bencana 2015).

The high risk of flood disasters in Langsa City is because of its location at an elevation of 0–25 metres above sea level (masl) and it is mostly a low-lying alluvial plain. Moreover, the rainfall intensity of the area is categorised as high, reaching 2300 mm/year. These conditions cause the city to experience floods almost every year as evidenced in the five flood events reported between 2010 and 2019 (BNPB 2019).

### Procedure

The experiment was conducted in the Learning Innovation Laboratory at Universitas Samudra. The process was initiated through the development of the IVR Flood Evacuation Program using Unity 3D application. Unity 3D is an application used to develop multi-platform games that are designed to be easy to use. It consists of several features, namely rendering, asset tracking, platform, asset store, physisc. This application has cross-platform advantages that can be used on various platforms such as Windows, Linux, Mac OS, Android and iOS. Unity 3 D also has many features that can be used to develop games.

Moreover, the equipment used include Oculus Quest 2, a VR headset and desktop software with an Intel i-7 processor and 32GB RAM. The experimental steps started with the introduction of IVR equipment and how to use the headset and touch controllers. Oculus Quest 2 is a wireless VR headset that functions to play games comfortably. It has a hand tracking feature that can be used to navigate menus and play games without touch controllers. It also has a camera tracking feature that allows users to walk around the room while wearing the headset without having to worry about bumping into objects.

The participants were asked to fill out a questionnaire consisting of eight items related to self-efficacy in facing flood disasters as well as to provide oral responses to three tests to assess the pre-training knowledge. After the training was completed, participants answered the same three questions orally to determine the knowledge after the training was conducted using the IVR Flood Evacuation programme.

### Measure

The effectiveness of the IVR Flood Evacuation Programme in disaster education was assessed by measuring the knowledge of best evacuation practices and self-efficacy in facing flood disasters. The focus was on the actions required before, during, and after floods using the questionnaire developed from the Disaster Facing Guide by the BNPB of Indonesia. Open-ended questions were used to avoid potentially biased answers from participants, and the same set of questions was answered orally before and after the training. Each answer to these questions has a score ranging from 1 to 4, where 1 means no knowledge and 4 means comprehensive knowledge in line with the scale developed based on the guidelines of the BNPB. Table 1 shows the aspects assessed, open-ended questions and the knowledge scale.

**TABLE 1:** Assessed knowledge aspects, open-ended questions for pre- and post-training and knowledge scale.

No.	Questions	Strong knowledge	Adequate knowledge	Weak knowledge	No knowledge
1	What do you do before a flood disaster occurs?	4 points for: (1) identifying the nearest evacuation route from home, (2) preparing a disaster readiness bag, (3) storing various important documents in a safe place	3 points for knowing to identify evacuation routes and prepare a disaster readiness bag	2 points for knowing to search for evacuation routes	1 point for knowing nothing
2	What do you do during a flood disaster?	4 points for: (1) evacuating to a higher place, (2) turning off all electrical networks, (3) not crossing swift currents, drainage channels, puddles and flooded areas, during evacuation	3 points for: (1) evacuating to a higher place, (2) turning off all electrical networks	2 points for knowing to evacuate to a higher place	1 point for knowing nothing
3	What do you do after a flood disaster?	4 points for: (1) returning home after receiving orders from authorities, (2) disposing of food contaminated by floodwater, (3) cleaning the living space and home environment to remove flood debris, (4) eradicating mosquito breeding sites	3 points for: (1) returning home after receiving orders from authorities, (2) disposing of food contaminated by floodwater	2 points for returning home after receiving orders from authorities	1 point for knowing nothing

No., number.

Self-efficacy is the belief of people in their ability to accomplish difficult tasks (Chichekian & Shore 2016; Liu et al. 2023). It has been discovered to have an influence on the behaviour of an individual (Newnham et al. 2017). The self-efficacy of the participants in dealing with flood disasters was measured using the General Self-Efficacy Scale (GSES) consisting of eight items (Benight et al. 1999). This assessment tool was preferred because of its high reliability value (alpha = 0.92) and wide application in several previous studies.

### Data analysis

The data obtained were analysed using *t*-tests through SPSS statistical software version 27.0.

### Ethical considerations

Ethical clearance to conduct this study was obtained from the Universitas Samudra Ethics Commission for Social Humanities at the Institute for Research and Community Service (No. 433/UN54.6/TU/2023).

## Results

The results obtained from the measurement showed that IVR was effective in enhancing the knowledge and self-efficacy of the participants regarding flood evacuation. This was indicated by the significant increase in knowledge after the training (*T*-test, *p* < 0.005) as presented in the mean and standard deviation data for each pre-test and post-test score recorded for best evacuation practices before, during and after a flood disaster as shown in Table 2.

**TABLE 2:** *T*-test results for knowledge level comparison.

Knowledge aspects	Pretest	Post-test
What do you do before a flood disaster occurs?	*M* = 1.80	*M* = 3.11
	s.d. = 0.45	s.d. = 0.42
	-	*p* < 0.005
What do you do during a flood disaster?	*M* = 1.96	*M* = 3.91
	s.d. = 0.42	s.d. = 0.28
	-	*p* < 0.005
What do you do after a flood disaster?	*M* = 1.87	*M* = 3.27
	s.d. = 0.45	s.d. = 0.44
	-	*p* < 0.005
**Total**	***M* = 5.62**	***M* = 10.29**
	**s.d. = 0.650**	***s.d.* = 0.626**
	-	***p* < 0.005**

s.d., standard deviation; *M*, mean.

Table 2 shows that the knowledge during a flood disaster has the highest score both before and after training compared to the other two components assessed. This was probably because most of the participants had experienced a flood disaster directly, leading to the provision of detailed answers to questions related to actions required during a flood. Moreover, a higher percentage of the participants live in Langsa City classified as an area with a very high risk of flood disasters in Indonesia. The *t*-test results for self-efficacy also showed a significant improvement after the training (*T*-test, *p* < 0.05) as presented in Table 3 and Table 4.

**TABLE 3:** *T*-test results for self-efficacy for pre-training and post-training.

Pretest self-efficacy	Post-test self-efficacy
*M* = 21.27	*M* = 34.11
s.d. = 1.643	s.d. = 1.153
*p* < 0.05	-

s.d., standard deviation; *M*, mean.

**TABLE 4:** Means and standard deviation for pretest and post-test self-efficacy.

No.	Questions	Pretest self-efficacy		Post-test self-efficacy	
		Means	s.d.	Means	s.d.
1	I am confident that I am able to effectively deal with a flooding emergency	2.62	0.490	4.33	0.477
2	Thanks to my resources, I know how to manage in a flooding emergency	2.82	0.387	4.22	0.420
3	I would be able to deal with a flood emergency even if the water level were critical and the speed of the water did not allow me to move easily	2.82	0.387	4.11	0.418
4	I would be able to cope with a flood emergency even if I found other people along the way	2.80	0.405	4.31	0.468
5	I would be able to cope with a flood emergency even if the exit were blocked and the water level did not allow me to open the doors and go out.	2.76	0.435	4.33	0.477
6	I would be able to deal with flood emergency even if I found objects that could injure me along the way	2.49	0.506	4.09	0.514
7	The consequence of a flooding emergency on my safety would be severe	2.64	0.484	4.40	0.495
8	I would be vulnerable during a flood	2.31	0.468	4.31	0.633

No., number; s.d., standard deviation.

## Discussion

The results showed that the scores for all three questions related to evacuation behaviour increased significantly. The average pretest score for the first question on what students should do before a flood occurs was found to be *M* = 1.80 on a scale of 4. This showed that most of the participants did not have a comprehensive knowledge of the actions to be taken before a flood and had only general knowledge such as identifying evacuation routes and the nearest points without any provision for disaster readiness bags and storage of important documents in a safe place.

A significant increase was recorded, *M* = 3.11 on a scale of 4 with significance (*P* < 0.005), after the training using IVR Flood Evacuation Programme. The improved scores could be attributed to the highly effective scenarios presented to represent the actions required before a disaster such as determining evacuation routes, storing important documents in a safe place, and preparing a disaster readiness bag containing supplies for independent living for 3 days. Moreover, the programme allowed the implementation of an experiential learning approach that engaged students directly in disaster risk reduction actions. This experiential learning is a teaching approach that places students in a physical or virtual environment in order to ensure the students truly learn by actively participating in practical scenarios (Gouramanis & MoralesRamirez 2020; Selby et al. 2012; Zavar & Nelan 2020). The method has been proved to be excellent in ensuring knowledge retention for students. However, there is a need for more regular disaster evacuation exercises or drills to maintain this knowledge. This is in line with the findings of Chittaro and Buttussi (2015) that repeated training using IVR can improve learning outcomes.

The pretest score for the answers on what to do during a flood was found to be *M* = 1.96. This score showed that most students did not have comprehensive knowledge of the actions to be taken during a flood according to the guidelines developed by the BNPB. A higher percentage of the students knew only general aspects such as evacuating to higher ground during a flood. However, the score increased significantly to *M* = 3.91 with significance (*P* < 0.005) after the training using the IVR Flood Evacuation Programme.

The increase in students’ knowledge was presumed to be because of the different scenarios presented through the implementation of the programme such as turning off the power before evacuation, not touching electrical equipment during evacuation, and staying away from swift currents or drainage channels when evacuating to the assembly point. This allowed the students to intuitively remember the experiences while using IVR, providing a long-term effect on their memory. The results reinforced previous research affirming that simulations or drills produced better outcomes and allowed longer knowledge retention compared to traditional learning methods (Feng et al. 2020; Gwynne et al. 2020; Khanal et al. 2022).

The pretest score on the questions related to the knowledge after a flood was recorded to be *M* = 1.87. This showed that the students had very little knowledge of the actions to be implemented after a flood as indicated by the response only indicating returning to the home after receiving orders from authorities. The low knowledge was because of the very limited training and socialisation by authorities regarding actions after a flood. Ideally, the students are expected to participate by taking several actions such as disposing of food contaminated by floodwater, cleaning homes and the surrounding environment, and collectively eradicating mosquito breeding sites within the community. Meanwhile, the post-test scores showed a significant increase to *M* = 3.27 on a scale of 4 after the training conducted using the programme.

The results further showed that the students had very high self-efficacy after participating in the training. It was discovered that the item related to ‘the consequence of a flooding emergency on my safety would be severe’ had the most significant increase (*M* = 4.40 on a scale of 5) compared to the other questions. This was presumed to be because of the presentation of scenarios that allowed the participants to experience a virtual flood disaster using the IVR program, thereby enhancing the perception of self-efficacy among the participants. Moreover, the training was observed to have the potential to foster disaster preparedness behaviour among the students (Chittaro & Sioni 2015; Feng et al. 2020).

Self-efficacy is an important variable in determining attitude and behavioural change (Feng et al. 2020). The results were observed to have supported the findings of several previous studies such as the study of Chittaro and Sioni (2015) that the application of interactive learning media like serious games was effective in increasing self-efficacy in disaster preparedness and motivating participants to take actions towards self-protection. Feng et al. (2020) also stated that IVR was efficient in increasing self-efficacy during earthquake hazards. Moreover, students with high self-efficacy were observed to have better disaster preparedness ability and made evacuation decisions because of the belief that the efforts could result in effective actions. This is in line with the observation of previous research that self-efficacy is an important indicator in individual decision-making (Liu et al. 2023; Newnham et al. 2017; Yip et al. 2013).

## Conclusion

In conclusion, this research aimed to develop and test the effectiveness of the IVR Flood Evacuation Programme to improve knowledge and self-efficacy regarding flood disasters. The IVR developed consisted of three scenarios including actions that need to be taken before, during and after a flood. The results showed that IVR was effective in significantly improving knowledge on the best evacuation practices and self-efficacy. However, regular flood disaster evacuation simulations are necessary to maintain the knowledge. This study recommends that training on flood disaster evacuation practices using IVR should be integrated into future disaster education programmes. This is important because transforming knowledge into action is the core of disaster education. Therefore, the IVR Flood Evacuation Programme has the potential to be applied as an engaging pedagogical tool for flood disaster evacuation training.
